# Supplementary material for: Decreased autophagy and fuel switching occur in a senescent hepatic cell model system
Source: Aging (Albany NY). 2020 Jul 26;12(14):13958–78. doi: 10.18632/aging.103740 (PMC7425478; doi:10.18632/aging.103740)
Supplement: Supplementary Methods [file aging-12-103740-s002..pdf]

SUPPLEMENTARY METHODS

RNAseq

Data analysis

Downstream analysis was performed using a combination of programs including STAR, HTseq, Cufflink and our wrapped scripts. Alignments were parsed using Tophat program and differential expressions were determined through DESeq2/edgeR. GO and KEGG enrichment were implemented by the ClusterProfiler. Gene fusion and difference of alternative splicing event were detected by Star-fusion and rMATS software

Reads mapping to the reference genome

Reference genome and gene model annotation files were downloaded from genome website browser (NCBI/UCSC/Ensembl) directly. Indexes of the reference genome was built using STAR and paired-end clean reads were aligned to the reference genome using STAR (v2.5) [1]. STAR used the method of Maximal Mappable Prefix(MMP) which can generate a precise mapping result for junction reads.

Quantification of gene expression level

HTSeq v0.6.1 was used to count the read numbers mapped of each gene. And then FPKM of each gene was calculated based on the length of the gene and reads count mapped to this gene. FPKM, Reads Per Kilobase of exon model per Million mapped reads, considers the effect of sequencing depth and gene length for the reads count at the same time, and is currently the most commonly used method for estimating gene expression levels [2].

Differential expression analysis

(For DESeq2 with biological replicates) Differential expression analysis between two conditions/groups (two biological replicates per condition) was performed using the DESeq2 R package (2_1.6.3). DESeq2 provide statistical routines for determining differential expression in digital gene expression data using a model based on the negative binomial distribution. The resulting P-values were adjusted using the Benjamini and Hochberg's approach for controlling the False Discovery Rate(FDR) [3]. Genes with an adjusted P-value <0.05 found by DESeq2 were assigned as differentially expressed [4].

(For edgeR without biological replicates) Prior to differential gene expression analysis, for each sequenced library, the read counts were adjusted by edgeR program package through one scaling normalized factor. Differential expression analysis of two conditions was performed using the edgeR R package (3.16.5). The P values were adjusted using the Benjamini & Hochberg method. Corrected P-value of 0.05 and absolute foldchange of 1 were set as the threshold for significantly differential expression [5].

The Venn diagrams were prepared using the function venn Diagram in R based on the gene list for different group.

Correlations

To allow for log adjustment, genes with 0 FPKM are assigned a value of 0.001. Correlation were determined using the cor.test function in R with options set alternative =”greater” and method = “Spearman”.

Clustering

To identify the correlation between difference, we clustered different samples using expression level FPKM to see the correlation using hierarchical clustering distance method with the function of heatmap, SOM(Self-organization mapping) and kmeans using silhouette coefficient to adapt the optimal classification with default parameter in R.

GO and KEGG enrichment analysis of differentially expressed genes

Gene Ontology (GO) enrichment analysis of differentially expressed genes was implemented by the clusterProfiler R package, in which gene length bias was corrected. GO terms with corrected P value less than 0.05 were considered significantly enriched by differential expressed genes [6].

KEGG [7] is a database resource for understanding high-level functions and utilities of the biological system, such as the cell, the organism and the ecosystem, from molecular level information, especially large-scale molecular datasets generated by genome sequencing and other high-through put experimental technologies (http://www.genome.jp/kegg/). We used clusterProfiler R package to test the statistical enrichment of differential expression genes in KEGG pathways.

PPI analysis of differentially expressed genes

PPI analysis of differentially expressed genes was based on the STRING database, which contained known and predicted Protein-Protein Interactions. For the species existing in the database(like human and mouse), we

constructed the networks by extracting the target gene lists from the database [8].

Fusion gene analysis

Fusion gene is refers to the two genes of all or part of the sequences perform fusion ,results of the chimeric gene, usually caused by reasons such as chromosome translocation and problem. We used Star-fusion(0.8.0) software analysis and detection of fusion genes [9].

Alternative splicing analysis

Alternative Splicing is an important mechanism for regulate the expression of genes and the variable of protein. rMATS(3.2.1) software was used to analysis the ASevent [6].

SNP analysis

We deal with the bam alignment results of each sample by using picard tools(v1.111) and samtools(v0.1.18), including reorder, sort, add head information, mark duplicates, local realignment around indels and base qulaity score recalibration [10]. Then we call snp by the tool HaplotypeCaller in GATK3.4 version [11]. Finally, we ues annovar to do SNP annotation against dbSNP database and some other database.

Differentially expressed gene annotation

TFCat and Cosmic database were used to annotate the differential expressed gene. TFCat is a curated catalogue of mouse and human transcription factors (TF) based on a reliable core collection of annotations obtained by expert review of the scientific literature [12]. COSMIC is a database designed to store and display somatic mutation information and related details which contains information relating to human cancers.

Data access

The high-throughput sequencing data from this study have been submitted to the GEO-NCBI under accession number GSE151806.

ACKNOWLEDGMENTS

We thank NovogeneAIT for technical supports and deep discussion.

REFERENCES

1. Dobin A, Davis CA, Schlesinger F, Drenkow J, Zaleski C, Jha S, Batut P, Chaisson M, Gingeras TR. STAR: ultrafast universal RNA-seq aligner. Bioinformatics. 2013; 29:15–21. https://doi.org/10.1093/bioinformatics/bts635 PMID:23104886

2. Mortazavi A, Williams BA, McCue K, Schaeffer L, Wold B. Mapping and quantifying mammalian transcriptomes by RNA-seq. Nat Methods. 2008; 5:621–28. https://doi.org/10.1038/nmeth.1226 PMID:18516045

3. Dillies MA, Rau A, Aubert J, Hennequet-Antier C, Jeanmougin M, Servant N, Keime C, Marot G, Castel D, Estelle J, Guernec G, Jagla B, Jouneau L, et al, and French StatOmique Consortium. A comprehensive evaluation of normalization methods for illumina high-throughput RNA sequencing data analysis. Brief Bioinform. 2013; 14:671–83. https://doi.org/10.1093/bib/bbs046 PMID:22988256

4. Anders S, Huber W. Differential expression analysis for sequence count data. Genome Biol. 2010; 11:R106. https://doi.org/10.1186/gb-2010-11-10-r106 PMID:20979621

5. Robinson MD, McCarthy DJ, Smyth GK. edgeR: a bioconductor package for differential expression analysis of digital gene expression data. Bioinformatics. 2010; 26:139–40. https://doi.org/10.1093/bioinformatics/btp616 PMID:19910308

6. Shen S, Park JW, Lu ZX, Lin L, Henry MD, Wu YN, Zhou Q, Xing Y. rMATS: robust and flexible detection of differential alternative splicing from replicate RNA-seq data. Proc Natl Acad Sci USA. 2014; 111:E5593–601. https://doi.org/10.1073/pnas.1419161111 PMID:25480548

7. Ogata H, Goto S, Sato K, Fujibuchi W, Bono H, Kanehisa M. KEGG: kyoto encyclopedia of genes and genomes. Nucleic Acids Res. 1999; 27:29–34. https://doi.org/10.1093/nar/27.1.29 PMID:9847135

8. Shannon P, Markiel A, Ozier O, Baliga NS, Wang JT, Ramage D, Amin N, Schwikowski B, Ideker T. Cytoscape: a software environment for integrated models of biomolecular interaction networks. Genome Res. 2003; 13:2498–504. https://doi.org/10.1101/gr.1239303 PMID:14597658

9. Haas BJ, Dobin A, Stransky N, Li B, Yang X, Tickle T, Bankapur A, Ganote C, Doak TG, Pochet N, Sun J, Wu CJ, Gingeras TR, et al. STAR-Fusion: Fast and Accurate Fusion Transcript Detection from RNA-Seq. bioRxiv. 2017. https://doi.org/10.1101/120295

10. Chepelev I, Wei G, Tang Q, Zhao K. Detection of single nucleotide variations in expressed exons of the human

genome using RNA-seq. Nucleic Acids Res. 2009; 37:e106.
https://doi.org/10.1093/nar/gkp507 PMID:19528076

11. McKenna A, Hanna M, Banks E, Sivachenko A, Cibulskis K, Kernytsky A, Garimella K, Altshuler D, Gabriel S, Daly M, DePristo MA. The genome analysis toolkit: a MapReduce framework for analyzing next-generation DNA sequencing data. Genome Res. 2010; 20:1297–303.
https://doi.org/10.1101/gr.107524.110
PMID:20644199

12. Fulton DL, Sundararajan S, Badis G, Hughes TR, Wasserman WW, Roach JC, Sladek R. TFCat: the curated catalog of mouse and human transcription factors. Genome Biol. 2009; 10:R29.
https://doi.org/10.1186/gb-2009-10-3-r29
PMID:19284633
