# Supplementary material for: Decreased autophagy and fuel switching occur in a senescent hepatic cell model system
Source: Aging (Albany NY). 2020 Jul 26;12(14):13958–78. doi: 10.18632/aging.103740 (PMC7425478; doi:10.18632/aging.103740)
Supplement: Supplementary Figures [file aging-12-103740-s003..pdf]

SUPPLEMENTARY FIGURES

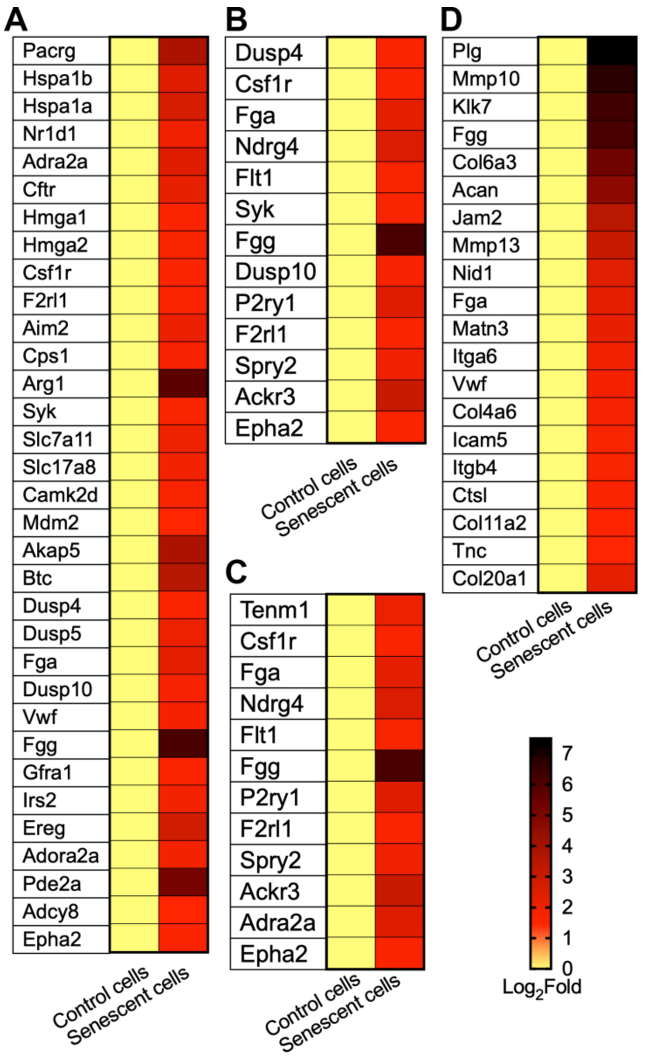

Supplemenatry Figure 1. RNAseq analysis of control and senescent AML12 cells for upregulated pathways. (**A**) Heat map showing genes regulating pathways: cAMP metabolic process (GO:0046058), positive regulation of tumor necrosis factor-mediated signaling pathway (GO:1903265), cellular response to unfolded protein (GO:0034620), regulation of insulin secretion involved in cellular response to glucose stimulus (GO:0061178), positive regulation of cellular senescence (GO:2000774), positive regulation of cell aging (GO:0090343), regulation of chemokine secretion (GO:0090196), interleukin-1 beta secretion (GO:0050702), interleukin-1 beta production (GO:0032611), bile acid biosynthetic process (GO:0006699), urea cycle (GO:0000050), regulation of superoxide anion generation (GO:0032928), L-glutamate transmembrane transporter activity (GO:0005313), Glutamate Binding, Activation of AMPA Receptors and Synaptic Plasticity Homo sapiens R-HSA-399721, Insulin receptor signalling cascade Homo sapiens R-HSA-74751, Signaling by Type 1 Insulin-like Growth Factor 1 Receptor (IGF1R) Homo sapiens R-HSA-2404192, IRS-mediated signalling Homo sapiens R-HSA-112399, and Signaling by Insulin receptor Homo sapiens R-HSA-74752. (**B**) Heat map showing genes regulating pathways: regulation of ERK1 and ERK2 cascade (GO:0070372), and Prolonged ERK activation events Homo sapiens R-HSA-169893. (**C**) Heat map showing genes regulating pathway: positive regulation of MAPK cascade (GO:0043410). (**D**) Heat map showing genes regulating pathway: interleukin-1 beta secretion (GO:0050702), interleukin-1 beta production (GO:0032611), extracellular matrix organization (GO:0030198), ECM-receptor interaction; Extracellular matrix organization Homo sapiens R-HSA-1474244.

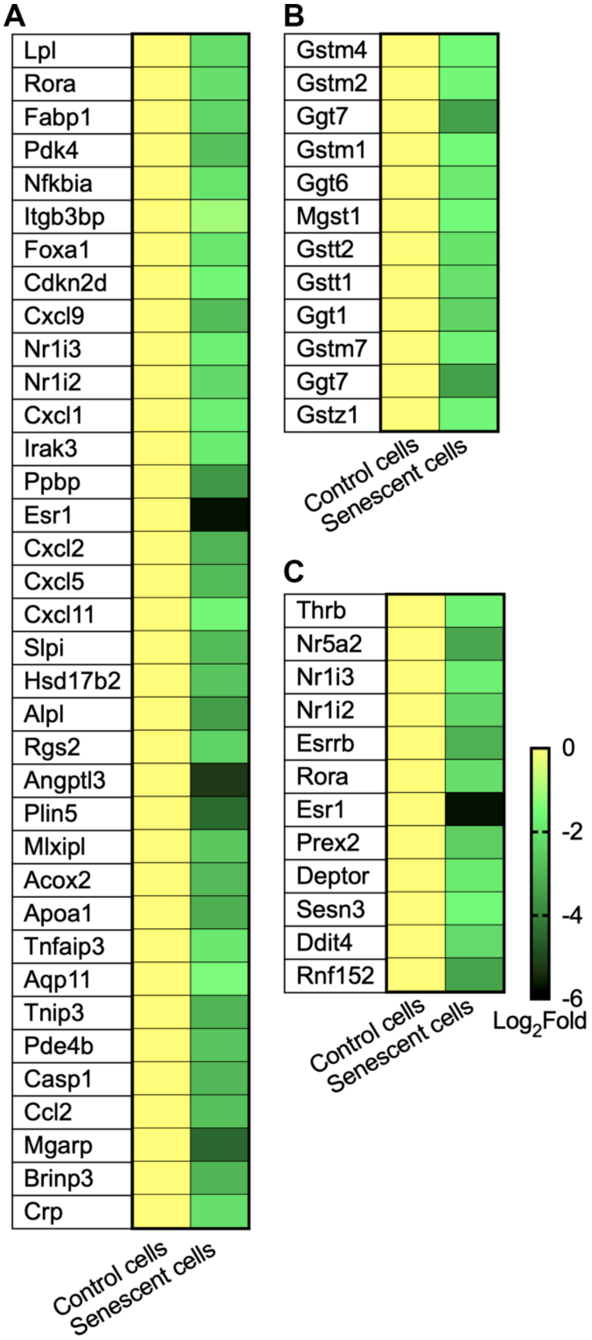

Supplementary Figure 2. RNAseq analysis of control and senescent AML12 cells for downregulated pathways. (**A**) Heat map showing genes regulating pathways: response to lipid (GO:0033993), negative regulation of lipase activity (GO:0060192), lipid homeostasis (GO:0055088), triglyceride homeostasis (GO:0070328), regulation of cholesterol homeostasis (GO:2000188), triglyceride catabolic process (GO:0019433), cellular response to lipid (GO:0071396), negative regulation of lipid storage (GO:0010888), and regulation of lipid biosynthetic process (GO:0046890). (**B**) Heat map showing genes regulating pathways: Glutathione metabolism, Glutathione conjugation Homo sapiens R-HSA-156590, Glutathione synthesis and recycling Homo sapiens R-HSA-174403, glutathione derivative biosynthetic process (GO:1901687), and glutathione metabolic process (GO:0006749). (**C**) Heat map showing genes regulating pathways: Nuclear Receptor transcription pathway Homo sapiens R-HSA-383280, and negative regulation of TOR signaling (GO:0032007).
